# Post-cardioversion Improvement in LV Function Defined by 4D Flow Patterns and Energetics in Patients With Atrial Fibrillation

**DOI:** 10.3389/fphys.2019.00659

**Published:** 2019-05-29

**Authors:** Lars Olof Karlsson, Hanna Erixon, Tino Ebbers, Ann Bolger, Carl-Johan Carlhäll

**Affiliations:** ^1^Department of Cardiology and Department of Medical and Health Sciences, Linköping University, Linköping, Sweden; ^2^Division of Cardiovascular Medicine, Department of Medical and Health Sciences, Linköping University, Linköping, Sweden; ^3^Center for Medical Image Science and Visualization (CMIV), Linköping University, Linköping, Sweden; ^4^Department of Medicine, University of California, San Francisco, San Francisco, CA, United States; ^5^Department of Clinical Physiology, Department of Medical and Health Sciences, Linköping University, Linköping, Sweden

**Keywords:** atrial fibrillation, atrial stunning, cardioversion, heart failure, LV function, 4D Flow CMR

## Abstract

**Background:**

Atrial fibrillation (AF) is a prevalent cause of cardiovascular morbidity, including thromboembolism and heart failure. Left ventricular dysfunction (LVD) detected in AF patients may be either precursor or consequence of the arrythmia. Successful cardioversion of chronic AF is often followed by a transient period of left atrial (LA) stunning, where depressed mechanical atrial contraction persists despite reinstitution of sinus rhythm. To determine if AF-associated LVD would improve with resolution of LA dysfunction, AF patients were examined immediately and 4 weeks after cardioversion to sinus rhythm. 4D flow cardiovascular magnetic resonance (CMR) assesses ventricular function according to the volumes and energetics of functional components of the LV volume. Previously, described 4D CMR markers of LVD include decreased volume and end-diastolic kinetic energy (KE) of the *Direct flow*, which is the portion of LV volume that passes directly from inflow to outflow in a single cycle. We hypothesize that impaired LV flow patterns and energetics will be found immediately after cardioversion during atrial stunning, and that those parameters will improve as atrial function returns.

**Methods:**

Ten patients with a history of AF underwent CMR 2–3 h (Time-1) and 4 weeks (Time-2), following electrical cardioversion to sinus rhythm. 4D phase-contrast velocity data and morphological images were acquired at a 3T CMR system. Using a previously evaluated method, pathlines were emitted from the LV end diastolic volume (LVEDV) and traced forward and backward in time until end-systole. The LVEDV was automatically separated into four functional flow components whose volume and KE were calculated.

**Results:**

Left atrial fractional area change increased over the follow-up period (*P* = 0.001), indicating recovery of LA mechanical function. LVEF increased between Time-1 and Time-2 (*P* = 0.003); LVEDVI did not change (*P* = 0.319). Over that interval, the ratios of *Direct flow*/LVEDV volume and KE increased (*P* = 0.001 and *P* = 0.003, respectively), while the ratios of *Residual volume*/LVEDV volume and KE decreased (*P* = 0.001 and *P* = 0.005, respectively).

**Conclusion:**

Post-cardioversion recovery of LA function was associated with improvements in conventional and 4D CMR markers of LV function. Flow-specific measures demonstrate the negative but potentially reversible impact of LA dysfunction on volume and energetic aspects of LV function.

## Introduction

Atrial fibrillation (AF) affects more than 3% of the population (Friberg and Bergfeldt, 2013). The condition is characterized by loss of organized atrial contraction and rapid, irregular heart rates. The condition carries increased risks of stroke, mortality, and development of heart failure (HF) (Benjamin et al., 1998; Cleland et al., 2003; Nieuwlaat et al., 2005; Bjorck et al., 2013). It is frequently difficult to determine the underlying mechanisms responsible for left ventricular dysfunction (LVD) discovered in patients with AF and whether it is an exacerbator or consequence of the arrhythmia. When no other cause of LVD is found, an arrhythmia-related cardiomyopathy is likely and may portend a better chance of improvement and influence treatment strategies (Gupta and Figueredo, 2014; Gopinathannair et al., 2015). Studies suggest that rhythm-restorative AF treatment such as catheter ablation may be associated with better outcomes than medical rate control, which allow heart rate irregularity and atrial dysfunction to persist (Hsu et al., 2004).

Several mechanisms underlying LVD in AF patients have been proposed, including sustained tachycardia, chronic heart rate irregularity, the loss of organized atrial contraction (Ling et al., 2012; Gupta and Figueredo, 2014; Ling et al., 2016; King et al., 2017), and the long-term impact of and potential for resolution of LVD are being actively studied. The presence of LVD is generally assessed with left ventricular ejection fraction (LVEF), which is volume-based and load dependent. Left ventricular dysfunction can also be sought using other methods, assessing alternative aspects of LV function, as for example flow-based measures from 4D cardiovascular magnetic resonance (CMR), which focus on intracardiac flow organization and efficiency (Dyverfeldt et al., 2015).

Four-dimensional flow CMR can assess the volume and kinetic energy (KE) of functional components of the left ventricular (LV) volume. The volume and KE at end-diastole of the portion of LV inflow which passes directly to outflow (the *Direct flow* component) reflect left atrial (LA)-ventricular coupling, and decreases in them have been proven to be markers of LVD in idiopathic dilated and ischemic cardiomyopathy with or without left bundle branch block (Zajac et al., 2018). Changes in other components, such as the LV *Residual volume*, also correlate with depressed LV function (Svalbring et al., 2016). The 4D flow CMR method is challeging in patients with AF, as irregular cycle lengths interfere with data acquisition and quality. However, the immediate post-cardioversion phase, when the electrical rhythm is normal but the mechanical function of the atrium remains depressed (Cibis et al., 2017), provides an excellent opportunity to study the effect of reduced LA function on LV function, and compare this with LV function after LA function has recovered.

We hypothesized that LVD would be identified in AF patients immediately after cardioversion using flow-based parameters of flow component volume and KE and that those parameters would improve toward normal values as LA mechanical dysfunction is resolved.

Therefore, the aim of this study was to use the volume and energetics of 4D blood flow components to assess changes in LV function in patients with a modest burden of AF immediately after conversion, when the atrium is stunned, and 4 weeks after cardioversion to normal sinus rhythm, when atrial mechanical contraction is restored.

## Materials and Methods

### Study Population

A total of 18 patients were included in the study. The regional ethics committee approved the study (Registration Number 2014/414-31, final decision 2014-12-11) and all patients gave written informed consent prior to participation in the study. The patients were recruited from the department of cardiology, Linköping University Hospital, Sweden. Patients with persistent AF lasting more than 3 weeks prior to cardioversion and scheduled for elective electrical cardioversion were included. Exclusion criteria were: (1) uncontrolled hypertension (>160/95 mmHg), (2) LV ejection fraction <40%, (3) age >80 years, (4) previous ablation or heart surgery, (5) more than mild to moderate valvular disorder of the aortic or mitral valve, (6) more than moderate dilatation of the left ventricle (LVEDVI <116 ml/m2 for males, < 101 ml/m2 for females), (7) contraindications for MRI-examination, and (8) recurrent AF occurring within the 4 weeks following the initial cardioversion that was not re-converted to sinus rhythm within 48 h. Eight patients were excluded, four due to recurrence of AF and four due to inadequate data quality.

### Data Acquisition

All 18 patients underwent a examination on a clinical 3 T CMR system (Philips Ingenia, Philips Medical Systems, Best, Netherlands) two to three h after the cardioversion (Time-1). Four weeks after the cardioversion (Time-2), a second CMR examination was performed on 14 patients. During each CMR examination, time-resolved, three-directional velocity data (4D flow MRI) in a volume encompassing the LA and LV were acquired along with morphological 2D bSSFP images in three standard long axis (LAx) orientations (two-, three-, and four-chamber) and two stacks of short axis (SAx). One SAx image stack was acquired prior to the 4D flow scan, and the other after the 4D flow scan. The 4D flow data were acquired during free-breathing using respiratory gating, and with interleaved three-directional flow encoding and retrospective, vector cardiogram controlled cardiac gating, thus covering the whole cardiac cycle. The arrhythmia rejection mode was activated in order to exclude possible irregular cardiac cycles. General acquisition parameters for the 4D flow data were: VENC: 120 cm/s; Repetition Time, 5.1 ms; Echo Time, 3 ms; Flip Angle, 5°. The spatial resolution was 2.8 × 2.8 × 2.8 mm^3^. Parallel imaging (SENSE) with a reduction factor of three was used to decrease the scan time. In order to further reduce scan time, two rows of k-space, where acquired for each scan segment per cardiac cycle (k-space segmentation factor = 2), resulting in an acquired temporal resolution of 40.8 ms. After the acquisition, the 4D flow data were reconstructed into 40 time frames on the scanner using a temporal sliding window with individual stretching of each RR-interval. Data were corrected for concomitant gradient field effects on the scanner.

The morphological bSSFP images were acquired using the following settings: Echo Time, 1.4 ms; Repetition Time, 2.8 ms, Flip Angle, 45°. The bSSFP images were reconstructed into 30 time frames with a slice thickness of 8 mm. SAx images were reconstructed with a pixel size of 0.9 × 0.9 mm^2^ and the LAx images 0.83 × 0.83 mm^2^.

### Data Analysis

The “Linköping 4D Flow Tool” was used to post–process and analyze the 4D flow MRI data (Eriksson et al., 2010). In this software, the 4D flow data were corrected for background phase errors by the use of a fourth order polynomial fitted to the static tissue surrounding the heart. The data were also corrected for phase wraps by the use of all temporal information in each voxel. After the data correction steps the flow data were exported in two formats, Matlab format for further analysis and a format compatible with commercial visualization software EnSight (CEI Inc., Research Triangle Park, NC, United States).

The LV flow components were analyzed using a previously evaluated method (Eriksson et al., 2010). The LV was segmented from the SAx-stack at times of end-diastole (ED) and end-systole (ES) by using Segment (v1.9 R3701, Medviso AB, Lund, Sweden). The two-, three-, and four-chamber LAx images provided additional aid in orientation and defining the ES and ED timeframes. The ED timeframe was set one timeframe after the closure of the mitral valve and with the aortic valve still closed. The ES timeframe was set one timeframe before the opening of the mitral valve with the aortic valve closed. The LV volumes at ES and ED were used to calculate LVEF and LVEDVI. The timeframe defining the onset of late diastole was identified from the LV volume curve as the end of the early filling phase.

Pathlines, which represent the course of virtual blood particles, were emitted at ED from an isotropic grid filling the end diastolic LV segmentation. Each pathline represented a volume of blood corresponding to the density of the grid 2.8 × 2.8 × 2.8 mm^3^. To measure pathlines’ trajectories during diastole, pathlines were traced backward in time in the reversed velocity field, which is like asking each pathline: Where did you come from? To measure trajectories during systole the pathlines were traced forward, which is like asking: Where are you going? By merging these backward and forward trajectories, the full EDV could be traced throughout the cardiac cycle from onset diastole to end systole. The ES segmentation was used to determine the pathlines’ location inside (retained) or outside (ejected) the LV at end-systole. All pathlines were then divided into four different components (Bolger et al., 2007; Eriksson et al., 2010):

*Direct Flow* (*DF*): Pathlines that enter and leave the LV during the analyzed cardiac cycle.

*Retained Inflow* (*RI*): Pathlines that enter the LV during diastole of the analyzed cardiac cycle and remain in the LV during the subsequent systole.

*Delayed Ejection Flow* (*DEF*): Pathlines that start within the LV and leave during the systole of the analyzed cardiac cycle.

*Residual Volume* (*RV*): Pathlines that start within the LV and remain within the LV at the end of systole.

The volume and KE were calculated from the particle traces assigned to each flow component, as previously described (Eriksson et al., 2011).

Conventional portions of LV volume including LV inflow, LV stroke volume and non-ejected LV volume were calculated from the flow-based components as follows:

LV inflow = *Direct flow* + *Retained inflow*

Stroke volume = *Direct flow* + *Delayed ejection flow*

Non-ejected volume = *Retained inflow* + *Residual volume*

The total LV inflow was calculated for the entire diastolic period, and late diastolic inflow was calculated over the late diastolic period.

Pathlines from all the subjects were visualized in EnSight (CEI Inc., Research Triangle Park, NC, United States). The overall pattern of flow within the LV throughout diastole, including the four flow components, was assessed by visual inspection by two investigators in consensus. The investigators had 1 and 20 years of experience in cardiac imaging, respectively.

In order to ascertain the quality of the acquired data, the differences in LV inflow and outflow were assessed for each study. Patients were excluded if the LV inflow versus outflow discrepancy was more than 15%. Flow pathlines were visualized in EnSight (CEI Inc., Research Triangle Park, NC, United States) and inspected for aberrant traces leaving the cardiac confines which would indicate impaired data quality.

The LA fractional area change (FAC) reflects the extent of atrial contraction. The largest and smallest LA areas were identified and measured twice from the 4-chamber long axis slice using Segment. The mean of the two measurements was used to calculate the LA FAC:

LA FAC = (largest LA area – smallest LA area) × 100/largest LA area

### Statistical Analysis

To test the normal distribution assumption of the samples the Kolmogorov–Smirnov test was used. Data are presented as mean ± 1 standard deviation (SD). Statistical comparisons were made with paired students *t*-tests. Statistical significance was set at *p* < 0.05. Since the number of included patients were rather few, we also performed a non-parametric analysis (Wilcoxon signed-rank test). This analysis revealed the same pattern of significance (data not shown) All statistical comparisons were made in Statistica (v12, Statsoft Inc., Tulsa, OK, United States).

## Results

Demographic and clinical patient data are provided in Table 1. The subjects included 1 female and 9 males aged 67 ± 7 years. At Time-1, 4 of 10 patients had a LVEF below 50%. Heart rate was slightly higher at Time-1 compared to Time-2 (61 ± 6, range 53–71, vs. 56 ± 6, range 48–65, *p* = 0.003). All patients were receiving beta-blocker therapy; all received angiotensin converting enzyme inhibitor/angiotensin II receptor blocker and/or calcium antagonists. The medication remained the same between Time-1 and -2. In terms of the previous burden of AF, seven out of ten patients had only experienced one episode of AF and the remaining three patients had experienced 2-3 episodes. The duration of the last AF episode was approximately one month in five of the patients and 2-3 months in the remaining five patients. For a more thorough description regarding clinical background data, please see Appendix.

**Table 1:** Patient demographic and clinical data.

	Time-1	Time-2	*P* value
Age (years)	67 ± 7		
Gender F/M	1/9		
Weight (kg)	92 ± 13		
Height (cm)	182 ± 10		
Heart rate (bpm)	61 ± 5 Range 53–71	56 ± 6 Range 48–65	0.003
*Blood pressure (mmHg)*			
Systolic	137 ± 17 Range 113–160		
Diastolic	87 ± 14 Range 68–100		
*Medication*			
Beta blockers	10/10		
ACE-inhibitors or ARB	8/10		
Calcium antagonist	7/10		
Diuretics	4/10		
Lipid-lowering drugs	4 / 10		
Anticoagulation with Warfarin or DOAC	10/10		


*ACE, angiotensin converting enzyme; ARB, angiotensin II receptor blocker; DOAC, direct oral anticoagulant.*

### Left Atrial Size and Mechanical Function

**FIGURE 1:**
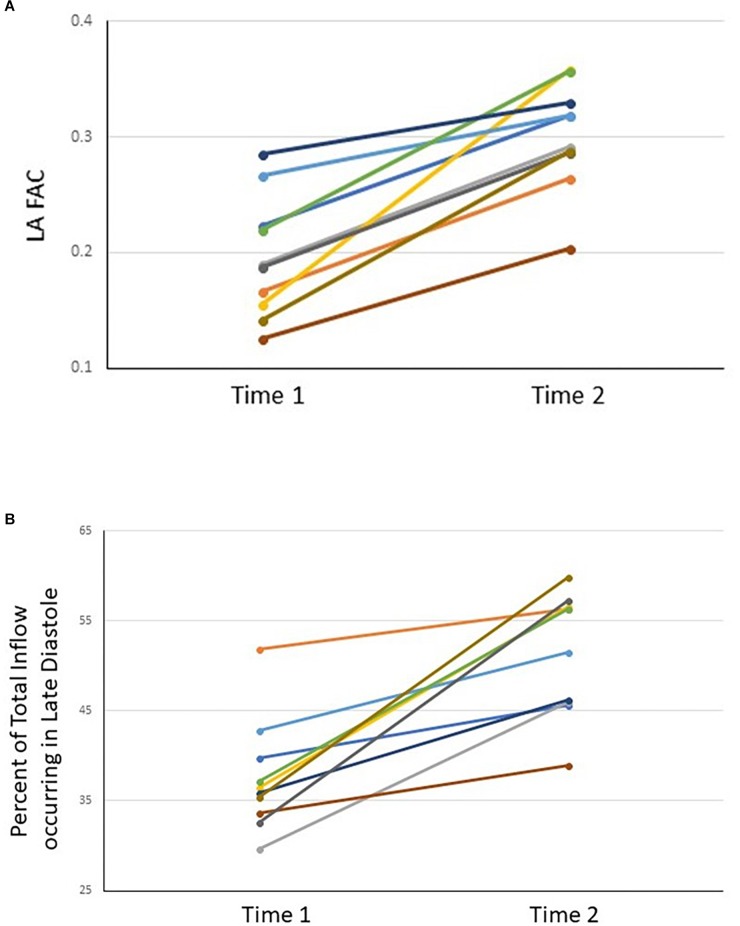
LA FAC **(A)** and percent of total inflow occurring in late diastole **(B)** for each subject at Time-1 and Time-2.

The maximal area of the left atrium did not show any significant change between Time-1 and Time-2 (*p* = 0.081), whereas the minimum area decreased over that interval (*p* = 0.008; Table 2). The LA FAC and the percentage of total inflow volume occurring in late diastole increased from Time-1 to Time-2 (*p* < 0.001 and *p* < 0.001, respectively; Tables 2, 3 and Figure 1). Six of ten patients demonstrated at least some features indicating coordinated atrial contraction on the immediate post-cardioversion study.

**Table 2:** Left atrial and ventricular sizes and function.

	Time-1	Time-2	*P* value
*Left atrium:*			
Maximum area (cm^2^)	36 ± 6	32 ± 6	0.081
Minimum area (cm^2^)	30 ± 6	22 ± 5	0.008
Fractional area change (%)	20 ± 5	30 ± 5	<0.001
*Left Ventricle:*			
LVEDVI (ml/m^2^)	85 ± 13	88 ± 13	0.319
LVESVI (ml/m^2^)	40 ± 11	34 ± 8	0.011
LVEF (%)	53 ± 8	61 ± 5	<0.001
Cardiac output (l/min)	4.7 ± 0.5	5.1 ± 0.8	0.138


*LVEDVI, left ventricular end diastolic volume index; LVESVI, left ventricular end systolic volume index; LVEF, left ventricular ejection fraction.*

**Table 3:** Change in inflow and stroke volume.

		Time-1	Time-2	*P* Value
*Volume:*				
	Total Inflow, ml	82 ± 9	94 ± 18	0.004
	% of total inflow volume occurring in late diastole	38 ± 6	52 ± 7	<0.001
	Stroke volume, ml	79 ± 9	93 ± 17	0.004
	Non-ejected Volume, ml	89 ± 25	74 ± 18	0.006
*KE at ED:*				
	Total Inflow, mJ	0.32 ± 0.1	1.1 ± 0.5	0.001
	% of total inflow KE occurring in late diastole	43 ± 10	59 ± 16	0.027
	Stroke Volume, mJ	0.33 ± 0.0	1.2 ± 0.5	<0.001
	Non-ejected Volume, mJ	0.2 ± 0.1	0.3 ± 0.2	0.049


### LV Size and Function

The LVEDVI did not change significantly from Time-1 to Time-2 (*p* = 0.319), whereas the LV end systolic volume index decreased (*p* = 0.011) (Table 2). In comparison to Time-1, the volumes of the total inflow and stroke volume increased by Time-2 (Table 3). The cardiac output did not change (*p* = 0.138).

**FIGURE 2:**
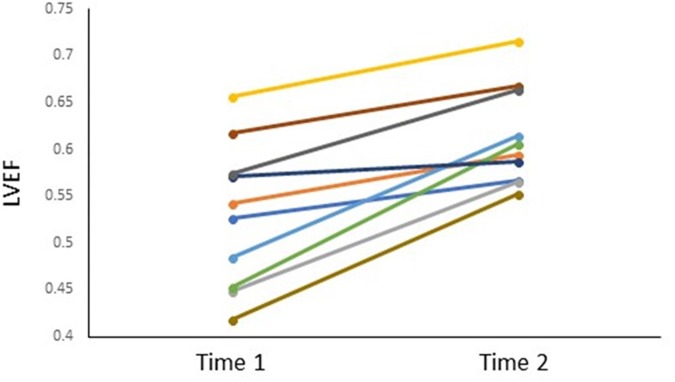
LVEF for each subject at Time-1 and Time-2.

Left ventricular ejection fraction increased from Time-1 to Time-2 (53 ± 8, 61 ± 5, respectively; *p* < 0.001). On an individual basis, the LVEF improved in all subjects over that interval (average change, 8% points, range 4 to 15; Figure 2). Four of ten subjects had an LVEF less than 50% at Time-1; the greatest increases in LVEF by Time-2 were seen in those four subjects (12, 13, 15, and 14% points, respectively).

### Flow Data Quality Assurance

Visual inspection of the 4D flow datasets did not reveal excessive aberrant pathlines. The difference between the measured inflow and outflow volumes for all patients was 4 ± 4 ml (range, 0.3 to 14 ml), representing 5 ± 4% (range, 0.3 to 15%) of the inflow volume.

### Left Ventricular Flow Components

**FIGURE 3:**
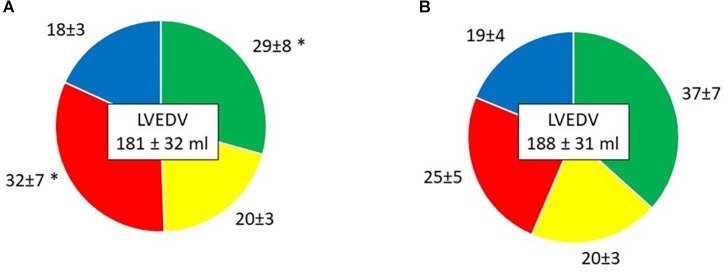
Volume of the four LV flow components in percentage of LVEDV, presented as mean ± SD. **(A)** Time-1; **(B)** Time-2. Green, *Direct flow*; Yellow, *Retained inflow*; Red, *Residual volume*; Blue, *Delayed ejection flow*. ^∗^*P* < 0.001 vs. Time-2.

**FIGURE 4:**
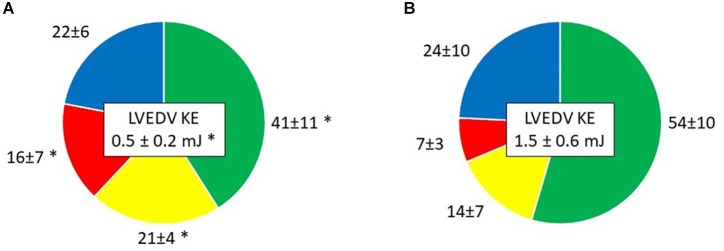
Kinetic energy (KE) at end diastole of the four LV flow components as a percent of LVED KE, presented as mean ± SD in **(A)** Time-1; **(B)** Time-2. Green, *Direct flow*; Yellow, *Retained inflow*; Red, *Residual volume*; Blue, *Delayed ejection flow*. ^∗^*P* < 0.05 vs. Time-2.

*Direct flow* volume and KE ratios increased from Time-1 to Time-2 (*p* < 0.001 and *p* = 0.002, respectively; Table 4 and Figure 3, 4). Over the same interval, the volume and KE ratios of the RV decreased (*p* < 0.001 and *p* < 0.001, respectively). The improvement in atrial contraction after cardioversion was reflected in the increase of the late diastolic contribution of volume and KE to the total inflow from Time-1 to Time-2 (*p* = 0.004; Table 3).

**Table 4:** LV flow component volume and KE ratios.

	Time-1	Time-2	*P* value
*Component volume ratio (% of LVEDV)*			
*Direct flow*	29 ± 8	37 ± 7	<0.001
*Retained inflow*	20 ± 3	20 ± 3	0.535
*Delayed ejection flow*	18 ± 3	19 ± 4	0.638
*Residual volume*	32 ± 7	25 ± 5	<0.001
*Component KE ratio (% of LVEDV KE)*			
*Direct flow*	41 ± 11	54 ± 10	0.002
*Retained inflow*	21 ± 4	14 ± 7	0.024
*Delayed ejection flow*	22 ± 6	24 ± 10	0.477
*Residual volume*	16 ± 7	7 ± 3	<0.001


*LVEDV, end diastolic volume measured from flow data; KE, kinetic energy measured at end diastole.*

Flow components were combined to evaluate the Stroke volume and the non-ejected volume. The volume and KE at ED of the stroke volume increased from Time-1 to Time-2 (*p* = 0.004 and *p* < 0.001, respectively). The non-ejected LV volume decreased in volume but increased in KE at ED from Time-1 to Time-2 (*p* = 0.006 and *p* = 0.049).

### LV Blood Flow Patterns

**FIGURE 5:**
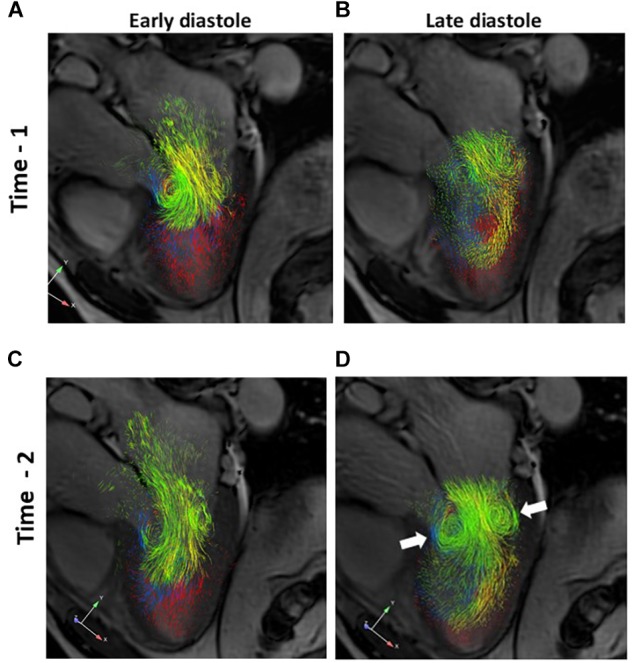
**A–D.** Three dimensional pathline visualization of diastolic LV flow components, projected on a two dimensional 3-chamber long axis slice for orientation. Images from a representative subject are shown. Early and late diastolic flows are shown for Time-1 (**A** and **B**, respectively) and for Time-2 (**C** and **D**, respectively). Note the more robust late diastolic inflow and better defined late diastolic submitral vortex ring (**D**, arrows) at Time-2 compared to Time-1. Green, *Direct flow*; Yellow, *Retained inflow*; Blue, *Delayed ejection flow*; Red, *Residual volume*.

Visual inspection of the overall diastolic flow within the LV was performed using three dimensional images to compare Time-1 to Time-2 for each subject. Early and late phases of diastole were considered. Immediately after cardioversion, there was a relative paucity of late diastolic versus early diastolic inflow in all patients. The submitral vortex ring was poorly organized in late diastole at Time-1. In comparison, by Time-2 the late diastolic inflow was more robust in all subjects, and comparable to early inflow. The late diastolic submitral vortex ring appeared more organized, with more pathlines involved in the rotational flow (Figure 5).

## Discussion

Patients with AF are heterogeneous in terms of etiology, duration and concomitant ventricular and atrial remodeling. Each of those factors influence the individual patient’s risk of AF recurrence and thromboembolism and may influence their treatment. Initial evaluation with echocardiography is routine for patients with AF, and many patients are found to have depressed LV systolic function even in the absence of clinicalHF. Atrial fibrillation and HF share many risk factors (Ling et al., 2016), but AF in and of itself may cause LVD. In the absence of another explanation for LVD, this is considered an arrhythmia-induced cardiomyopathy (AICM) (Gopinathannair et al., 2015).

Definition of the mechanisms and reversibility of AICM may inform important management decisions for patients in AF, including the decision to undergo invasive catheter ablation (Hsu et al., 2004; Khan et al., 2008). Sustained rapid heart rates, as may occur in AF, can cause depression of intrinsic myocardial contractility due to depletion of myocardial energy stores and abnormal myocardial calcium handling (Gupta and Figueredo, 2014). The degree of LVD is related to the rate and duration of tachycardia, and recovery of systolic function generally follows restoration of normal sinus rhythm. A second mechanism relevant to AF is the impact of chronic irregularity of the heart rhythm. (Ling et al., 2012).

The consequences and reversibility of LVD in AF has been carefully studied (Gupta and Figueredo, 2014; Gopinathannair et al., 2015). Improvement in LVEF following electrical cardioversion has been documented (Viswanathan et al., 2001; Wozakowska-Kaplon and Opolski, 2004; Zimmermann et al., 2015) and attributed to elimination of tachycardic episodes as well as Frank-Starling mechanisms (Xiong et al., 1995; Emilsson and Wandt, 2000). Improvements in LVD have been demonstrated in some but not all randomized trials of catheter ablation of AF (Khan et al., 2008; MacDonald et al., 2011; Jones et al., 2013; Hunter et al., 2014; Di Biase et al., 2016); one explanation for the different findings has been the spectrum of the populations with respect to idiopathic versus non-AF etiology, and heterogeneity in the severity of LVD.

Sustained AF has been shown to affect the LV myocardium anatomically. In the CAMERA-MRI study, late gadolinium enhancement demonstrated LV fibrosis in some AF patients with idiopathic LVD; absence of LV fibrosis correlated with the degree to which LV function, measured with LVEF, improved after catheter ablation and maintenance of sinus rhythm (Prabhu et al., 2017; Prabhu et al., 2018). Demonstrable reversibility of these functional and anatomic manifestations of AF-related LVD after cardioversion underscores the need for improved detection and definition of the mechanisms linking AF and LVD.

The period immediately following cardioversion is known to be associated with delayed recovery in LA mechanical function, called atrial stunning. The physiological impact of atrial stunning differs from AF; the rhythm irregularity of AF is removed, and the mechanical function of the LA is depressed but not absent as in AF. Atrial contraction can be demonstrated as early as one h after cardioversion of AF, and has been shown to improve over time as sinus rhythm is maintained (Zimmermann et al., 2015). The loss of the “booster” function of the atrial contraction on filling is blamed for clinical symptoms in AF, particularly among patients with impaired diastolic function, and regaining it is an important rationale for conversion to sinus rhythm.

We studied a small group of patients over the 4 weeks following electrical cardioversion of AF to normal sinus rhythm. As expected, signs of atrial stunning were apparent at Time-1, while LA area fraction and late diastolic inflow volume and KE at ED improved by Time-2 as stunning decreased.

Conventional and novel, flow-based metrics of LV function were used to define the change in underlying LV function and remodeling from volume and energetic perspectives. These patients had a limited prior burden of AF, with known duration of less than three months and measured in weeks in several. Only four had a LVEF less than 50% at Time-1. Despite the group’s heterogeneity with respect to LV and LA dysfunction, there were significant improvements in all features of LV function, and every patient had some degree of improvement in every parameter. The four subjects with depressed LVEF saw the greatest improvements in LVEF and flow-based metrics.

Previously, the asymmetric redirection of streaming blood in the heart, which were assumed to have potential fluidic and dynamic advantages, were believed to result from the asymmetries and curvatures of the looped heart (Kilner et al., 2000). Our findings reveal the importance of the atrial contraction in directing blood flow in the left ventricle such that a large volume leaves the LV in the following systolic ejection, which is reflected in the large portion of *Direct flow*, and with maintained momentum, reflected in the high KE at end diastole in the ejected blood. This indicates the importance of the atrial contraction beyond increased LV filling and myocardial stretch.

Left ventricular ejection fraction is the most widely used descriptor of LV systolic function, despite its recognized imprecision and load-dependence. It reflects the contributions of multiple determinants of LV filling, ejection, and systemic resistance. Flow-based parameters focus on different aspects of LV function than LVEF, including the dynamics of volume distribution and energy changes within the cardiac chambers (Eriksson et al., 2010, 2011; Svalbring et al., 2016). In AF and atrial stunning, the changes in late diastolic inflow can be expected to change the intracavitary flow routes and structures, and the “booster” contribution of the volume and energy bolus resulting from atrial contraction.

The late diastolic inflow volume and KE at ED increased by Time-2, confirming the impact of the recovered atrial mechanical function. The submitral vortex, which is normally well developed in late diastole, improved by Time-2. Flow parameters demonstrated an improvement in the amount and energy of the most efficient *Direct flow*, and the overall stroke volume. There was a corresponding decrease in the volume of the *Residual volume* component, and the non-ejected volume overall. The KE at ED of the non-ejected volume increased, indicating that the velocities in that volume increased, diminishing the potential for stasis, risk of thrombosis and inefficient filling and ejection. This flow analysis therefore, suggests that as the LV improves after cardioversion, there is a shift to more efficient ventricular function with less potential for stasis, even among patients with relatively normal LVEF at baseline.

The changes in flow-based parameters in this study are in line with prior studies. Compared to normal subjects, a reduced relative amount of *Direct flow* and increase in *Residual volume* has been demonstrated in patients with ventricular dysfunction. The Time-1 values among patients in this study are in line with the values measured by Eriksson et al (2013) in patients with mild to moderate LVD. The Time-2 *Direct flow* volume ratio (37%) is close to the values reported in subjects with normal cardiac function in previous studies (Eriksson et al., 2011; Stoll et al., 2018). Previous work has suggested that late diastolic inflow contains a larger percentage of *Direct flow* than the early inflow (Eriksson et al., 2011); the recovery of mechanical atrial contraction may have contributed to the observed increase in DF volume ratio at Time-2. The visible changes in diastolic flow patterns are also concordant with the observations by Eriksson et al (2011).

Further studies would be required to separate the relative impacts of atrial mechanical contraction and the phasic regularity of the heart rate on efficient LV ejection and flow management in normal and depressed ventricles. Flow-based analysis as applied here can provide a set of volume and energetic parameters that are complementary to conventional assessments and reflect important energetic features of the recovering ventricle.

### Limitations

The present CMR data relate to subjects in the supine position at rest and in sinus rhythm. Both the bSSFP and the 4D flow CMR data were acquired during end-expiration and are only representative for that phase in the respiratory cycle. Patient motion during the CMR scan may give rise to data mismatch: registration of data during the analysis aimed at alleviating this mismatch and data were formally assessed with quality control measures.

Patients with AF are heterogeneous due to variable cardiovascular substrate and the duration and severity of the condition. This small set of patients cannot represent the full spectrum of ventricular findings encountered in post-cardioversion patients. However, the key findings were based on paired intra-group comparison over time. 4D Flow CMR represents the average of velocities over a large number of heart beats, and hence, cannot assess beat to beat variations in LV flow. Marked irregularity of the heart rate as in AF would result in a non-representative “average cycle” and interfere with data quality. Only patients with persistent AF who had been successfully converted to sinus rhythm were included, and thus, had sinus rhythm during the presence of atrial stunning. The phase of atrial stunning is not expected to mimic flow conditions that would be seen in AF, where heart rate irregularity and absence of mechanical atrial contraction are seen.

## Conclusion

Loss of LA mechanical activity may contribute to LVD and HF in AF. During the period of LA stunning following cardioversion, flow-based measured parameters of volume and end-diastolic KE of *Direct flow* demonstrated impairment of LV function which improved with recovery of LA mechanical function by 4 weeks later. In addition, the volume and KE ratios of the *Residual volume*, which may contribute to ventricular inefficiency and stasis, were diminished at follow up. These 4D flow-specific measures may reflect novel features of the ventricular benefits of reinstitution of sinus rhythm in the AF patient, and emphasize the importance of the atrial contraction in efficient flow-based atrioventricular coupling.

## Ethics Statement

This study was carried out in accordance with the recommendations of Regional Ethical Review Board with written informed consent from all subjects. All subjects gave written informed consent in accordance with the Declaration of Helsinki. The protocol was approved by the Regional Ethical Review Board (Dnr 2014/414-31).

## Author Contributions

LK, AB, and CC conceived and designed the study and edited the manuscript critically. LK and HE recruited the patients. LK, HE, TE, and CC were involved in data collection. HE and CC analyzed the data. HE, AB, and CC interpreted the results and prepared the figures. HE drafted the manuscript. TE revised the manuscript. All authors read, approved the final manuscript and agreed to be accountable for all aspects of the work in ensuring that questions related to the accuracy of integrity of any part of the work are appropriately investigated and resolved.

## Conflict of Interest Statement

The authors declare that the research was conducted in the absence of any commercial or financial relationships that could be construed as a potential conflict of interest.
