# Supplementary material for: Post-cardioversion Improvement in LV Function Defined by 4D Flow Patterns and Energetics in Patients With Atrial Fibrillation
Source: Front Physiol. 2019 May 29;10:659. doi: 10.3389/fphys.2019.00659 (PMC6549517; doi:10.3389/fphys.2019.00659)
Supplement: Supplementary file 1 [file Table_1.docx]

| **Patient** | **Comorbidity** | **Mean HR during AF (BPM)** | **Mean HR during SR (BPM)** | **AAD at DC** |
| --- | --- | --- | --- | --- |
| **1** | Hypertension | 57 | 51 | BB, flecainide |
| **2** | Hypertension | 67 | 58 | BB |
| **3** | Hypertension | 87 | 50 | BB, cordarone |
| **4** | Hypertension, hyperlipidemia | 56 | 58 | BB |
| **5** | DM, Hypertension, stroke | 71 | 53 | BB, flecainide |
| **6** | Ischemic heart disease | 62 | 50 | BB |
| **7** | COPD, Hypertension | 81 | 71 | BB |
| **8** | DM, Previous gastric bypass | 83 | 57 | BB |
| **9** | Hypertension, possible stroke | 78 | 51 | BB |
| **10** | Hypertension | 138 | 67 | BB, dronedarone |

**Appendix**

**Baseline characteristics for included patients.** DM, Diabetes Mellitus; COPD, Chronic Obstructive Pulmonary Disease; AF, atrial fibrillation; BB, Beta blocker; HR, heart rate; BPM, beats per minute; AAD, Antiarrhythmic medication; DC, Direct Cardioversion
